# Comparison of a pediatric practice-based therapy and an interdisciplinary ambulatory treatment in social pediatric centers for migraine in children: a nation-wide randomized-controlled trial in Germany: “moma – modules on migraine activity”

**DOI:** 10.1186/s12887-021-02757-2

**Published:** 2021-06-30

**Authors:** Mirjam N. Landgraf, Florian Heinen, Lucia Gerstl, Christine Kainz, Ruth Ruscheweyh, Andreas Straube, Joerg Scheidt, Sabine von Mutius, Viola Obermeier, Ruediger von Kries

**Affiliations:** 1 Department for Neuropediatrics, Developmental Medicine and Social Pediatrics, LMU Center for Development and Children with Medical Complexity – iSPZ Hauner, Dr. von Haunersches Kinderspital, Kinderklinik und Kinderpoliklink der Ludwig Maximilians Universitat Munchen, Munich, Germany; 2 Department of Neurology, Ludwig-Maximilians-Universitat Munchen, Munich, Germany; 3 Hochschule Hof, Institute of Information Systems, Hof, Germany; 4 Ludwig-Maximilians-Universitat Munchen, Institute for Social Pediatrics and Adolescent Medicine, Munich, Germany

**Keywords:** Migraine, Children, Intervention, Interdisciplinary, Pediatric practice

## Abstract

**Background:**

Migraine is common in childhood, peaks in adolescents and persists into adulthood in at least 40% of patients. There is need for early interventions to improve the burden of disease and, if possible, reduce chronification. The aim of the project is to compare two types of ambulatory treatment strategies regarding their effect on headache days and quality of life in 6 to 11 year old children with migraine: 1) the routine care in pediatricians’ practices (intervention group A) and 2) a structured interdisciplinary multimodal intervention administered at social pediatric centers (intervention group B).

**Methods:**

The study is a nation-wide cluster-randomized study. Based on the postal codes the regions are randomly assigned to the two intervention-strategies. Children with migraine are recruited in the pediatric practices, as common outpatient-care in the German health-care system. Parents rate headache frequency, intensity and acute medication intake at a daily basis via a digital smartphone application specifically designed for the study. Migraine-related disability and quality of life are assessed every 3 months. Study duration is 9 months for every participant: 3 months of baseline at the pediatric practice (both groups); 3 months of intervention at the pediatric practice (intervention group A) or at the social pediatric center (intervention group B), respectively; 3 months of follow-up at the pediatric practice (both groups).

**Discussion:**

Results of the planned comparison of routine care in pediatric practices and interdisciplinary social pediatric centers will be relevant for treatment of children with migraine, both for the individual and for the health care system.

**Trial registration:**

The study was approved by the ethics committee at the Ludwig-Maximilians-University Munich (number 18–804) and was retrospectively registered on 27 April 2021 in the WHO approved German Clinical Trials Register (number DRKS00016698).

## Background

Migraine is a chronic disease often starting in childhood. The Barmer health insurance company dataset, which is covering 11% of all German policyholders, showed that in 2015 approximately 1% of all children received a diagnosis of migraine already at the age of primary school (6 to 11 years of age) [1]. In patients with migraine diagnosed at the age of 7 years, migraine persists into adulthood in 65% of the affected females and 21% of the affected males [2].

The Barmer insurance data also reveal that only half of the primary school children diagnosed with migraine got appropriate medication e.g. NSAID, but 2.1% were at least once in inpatient treatment for migraine. Only 6% of the affected children received an interdisciplinary therapy at a social pediatric center (unpublished data). Social pediatric centers are regional neuropediatric centers offering interdisciplinary treatment for children with chronic diseases. They belong to the customary care institutions in Germany.

It has repeatedly been shown that interdisciplinary treatment is effective in adult patients with migraine: e.g., in one study 42.5% of patients achieved a reduction of headache days of more than 50% even in the long term [3] and in another study, the number of headache days was significantly reduced from 13.8 ± 7.6 to 8.2 ± 6.2 [4]. Comparisons with standard basic care have shown superiority of multimodal treatment [5, 6].

Studies about the efficacy of multimodal treatment in young children with migraine do not exist so far. Children with migraine have a shorter migraine history and less comorbidities, such as other pain syndromes or psychiatric disorders, than adult migraine patients. Therefore, an early interdisciplinary multimodal treatment in young children may lead to an even better outcome in comparison to adults and may prevent the establishing of co-morbidities in the long term. Effective elements of treatment seem to include counselling on life style factors [7, 8] and psychotherapeutic [9] and physiotherapeutic measures [9, 10]. These interdisciplinary multimodal approaches are a distinguishing feature of German social pediatric centers.

Therefore, we designed an interdisciplinary multimodal intervention for treatment of pediatric migraine in social pediatric centers and a randomized controlled study to evaluate its effectiveness compared with standard basic pediatric care over a 9 months interval (elements of the interdisciplinary intervention: see 2.3. Study Design).

The aim of the presented study is to evaluate if an early intervention in young children at the time migraine has just started leads to a reduction of headache days and headache intensity as well as to a lower intake of analgesics and to an amelioration of quality of life.

## Methods/design

### Patient involvement

Children with migraine are treated in pediatric practices and social pediatric centers in Germany. As interdisciplinary multimodal treatment showed superior effects in the care of adults with migraine, we included pediatric, psychological and physiotherapeutic modules in the treatment of children with migraine in our clinical work at the social pediatric center in Munich. The satisfaction with this treatment of both children with migraine and their parents thereby ameliorated in a significant way. Based on this experience, we developed the patient-centered, structured, interdisciplinary multimodal intervention “moma – modules on migraine activity”, presented in this protocol. Nevertheless, pediatricians in practice are the standard basic pediatric care for children with migraine in Germany and are often very effective. Thus, we chose treatment in pediatric practices as comparative intervention strategy in our study design. Another aspect of patient involvement in the study design was that, to our clinical experience, the acceptance of treatment support via digital applications is high among parents in the neuropediatric field. Therefore, we developed the “moma app” for smartphones to facilitate the documentation of headache symptoms for the parents of participating children with migraine. The results of the study will be disseminated not only in scientific journals but also in public media to attain transparency for patients and their parents about the effectiveness of different treatment strategies for children with migraine.

### Selection of sample

Children between 6 and 11 years of age with migraine with a statutory health insurance (approx. 90% of the population) and whose parents consent to participation are eligible for the study. Children are recruited by their pediatricians in practice. The pediatrician diagnoses migraine according to the International Classification of Headache Disorders 3rd edition (ICHD-3) [11], with the help of a standardized, electronic migraine checklist that was developed for the study.

Inclusion criteria are as follows:

- Age 6 to 11 years
- Diagnosis of migraine (according to the International Classification of Diseases ICD-10, classification numbers G43.x), based on the International Classification of Headache Disorders (ICHD-3) [11] adapted to children (allowing shorter headache duration of 2 to 72 h, and bilateral instead of unilateral headache)
- History of migraine for at least 3 months
- At least 3 headache days in the last 3 months
- Migraine prophylactic medication (beta-blocker, amitriptyline, topiramate) allowed if kept constant for the duration of the study

Exclusion criteria are:

- Mental retardation (IQ < 70)
- Severe somatic or psychological, acute or chronic disease except headache
- Familiar hemiplegic migraine (genetically confirmed)
- Children will be excluded after the baseline phase if less than 3 headache days were present during the 3 months baseline phase

### Sample size calculation

The primary outcome was defined as a difference in reduction of headache days between the baseline and the follow-up period of ≥2 headache days in 12 weeks. The significance level α was set at 0.05 and the power at 0.9. Power size calculation with these parameters shows that a sample of *n* = 279 in the intervention group A (pediatric practice) and *n* = 507 children in the intervention group B (interdisciplinary treatment in social pediatric centers) is needed. For the sample size calculation an intra-class correlation coefficient (ICC) of 0.1 was assumed.

The estimated number for recruitment is based on an analysis of the Barmer insurance database of 2017 [1]. Approximately *n* = 270,000 statuarily insured children at the age of 6 to 11 years are likely to obtain a diagnosis of a primary headache disorder. About 70% of these see a pediatrician for primary care. As approximately 20% of pediatricians in practice use the website “PädExpert”, which provides the electronic migraine checklist, inclusion and exclusion criteria and access to the study platform, we calculated that *n* = 28,350 children per year are available for screening of migraine at the pediatric practices. We estimate that of these, 17.5% in fact receive a migraine diagnosis, resulting in *n* = 4961 children.

Germany was divided in 77 regions according to the postal codes, in a way that every postal code region contained at least one social pediatric center. The postal code regions were then randomized at a 2:1 ratio to intervention B and to intervention A regions. We estimated that in 75% of the intervention B regions at least one social pediatric center would be willing to participate in the study. We assumed that up to 20% of children will be excluded after the baseline phase or drop out later in the study: 1) because of more than 33% of missing data in the electronic headache diary during the baseline phase or 2) because of less than three documented headache days during the 3 months baseline or 3) because of more than 66% of missing data in the electronic headache diary during the follow-up period or 4) because they are lost to follow-up.

**Fig. 1:**
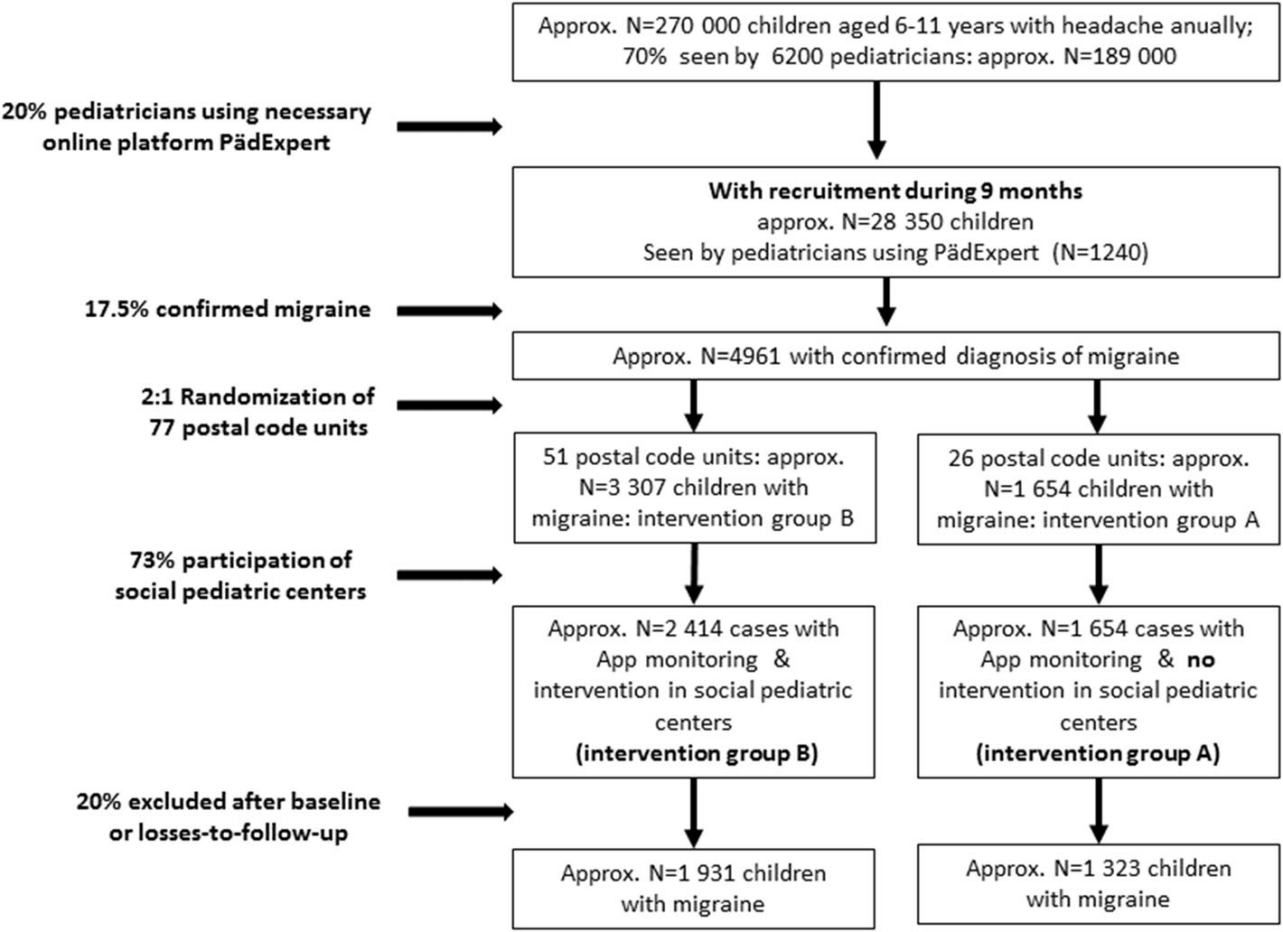
Flow chart of the expected number of children with migraine treated by the interdisciplinary multimodal intervention in the social pediatric centers (intervention group B) and in the pediatric practice (intervention group A)

As shown in the flow chart (Fig. 1) the actual percentage of regions where at least one social pediatric center participated (intervention group B) was 73% (only slightly lower than estimated). Because of an assumed further drop out of 20% after recruitment, the numbers expected were *n* = 1931 and *n* = 1323 for the intervention B (social pediatric centers) and intervention A (pediatric practice) group respectively. These numbers exceed the required numbers as estimated in the power calculation. We chose a broad safety margin because of uncertainties related to the COVID-19 pandemic.

### Study design

#### Overview (Fig. 2)

**Fig. 2:**
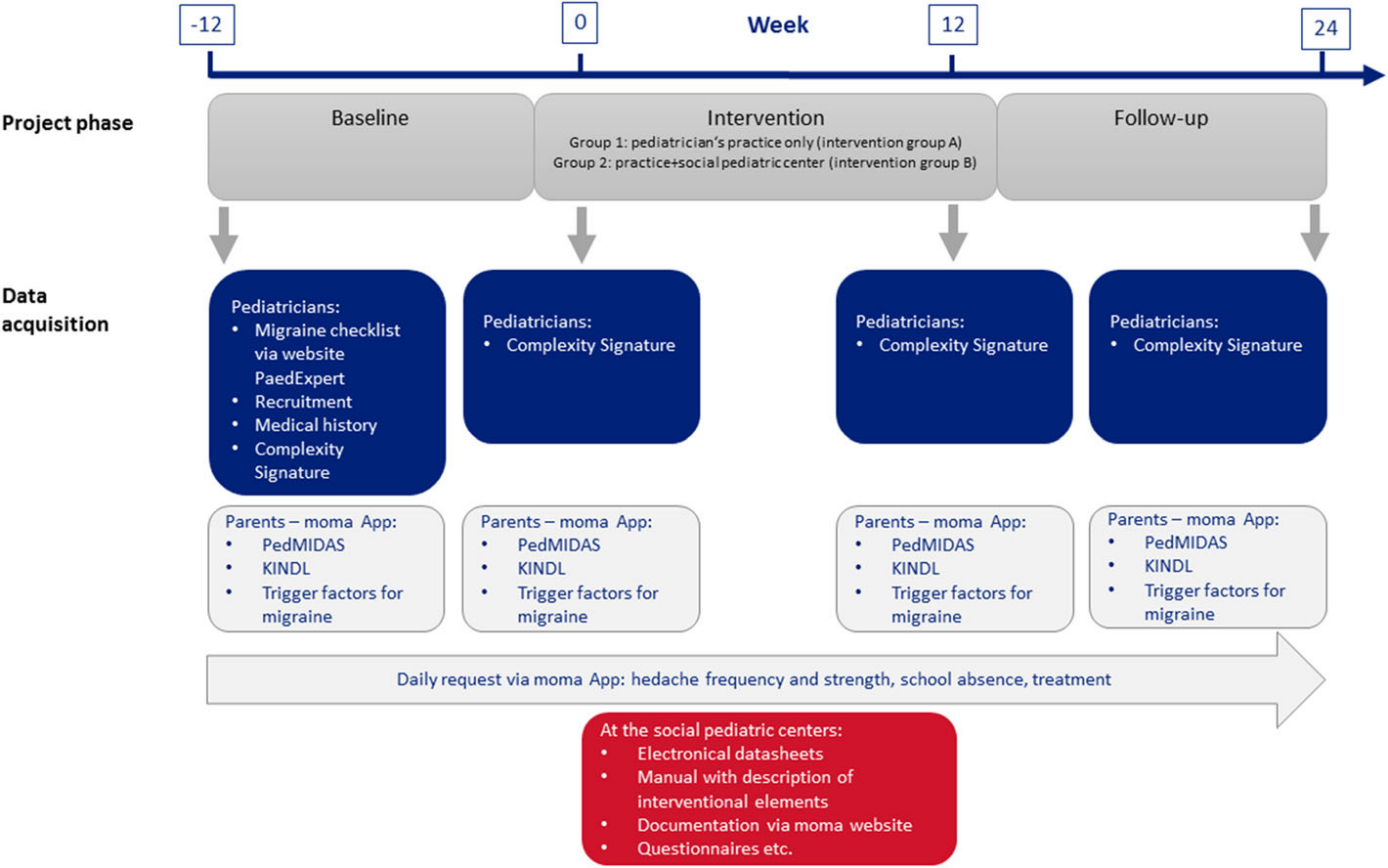
Study design, timelines and data acquisition

As described above, the study compares the pediatric practice treatment (intervention group A) and the interdisciplinary multimodal treatment (intervention group B). The timeline is illustrated in Fig. 2.

In both groups, children are diagnosed with migraine by their pediatrician in practice, included in the study and, if they have at least 3 days of headache in the baseline phase (week − 12 to 0), go on to the intervention phase (week 0–12). The intervention phase includes treatment at the pediatric practice for both groups, and treatment at the social pediatric center only for the interdisciplinary multimodal group (intervention group B). There, children are treated with the structured multimodal interdisciplinary intervention “moma” as described below. After the intervention the follow-up (week 12 to 24) is performed by the treating pediatrician in practice again.

Every participant has four study visits at the pediatric practice at time points − 12, 0, 12 and 24 weeks. The interdisciplinary multimodal intervention group has three additional study (intervention) visits at the social pediatric centers.

At the first visit, the moma web application generates a hashcode for pseudonymization of the patient, which is given to the parents by the pediatrician. The parents use the hashcode to log into the “moma App”, in which they document headache frequency, intensity and acute headache medication of their child on a daily basis.

#### Interdisciplinary multimodal intervention

The elements of the presented structured multidisciplinary intervention for children at the age of 6 to 11 years “moma” include:

1. pediatric elements such as exclusion of secondary causes and comorbidities by neurological examination and blood tests, and counselling regarding the bio-psycho-social model of pain,
2. physiotherapeutic elements such as examination of myofascial trigger points in the neck and shoulder muscles, education about the association of muscle tenderness and migraine and about individual exercise and self-administered stretching measures,
3. psychological elements such as evaluation of psychosocial circumstances, relevant psychological and behavioral factors for migraine, introduction of relaxation techniques and individual counselling to address the identified factors and improve self-efficacy.

The pathophysiological rationale for choosing these therapeutic elements is as follows:

According to epidemiological data, 45 to 85% of German adolescents with migraine (depending on migraine frequency) also suffer from muscle pain of neck or shoulders [9]. The pathophysiological explanation for this association is the concept of the trigemino-cervical complex (TCC) with its convergence of nociceptive afferents from cervical muscles to the caudal trigeminal nuclei [12]. As invasive or drug treatment is often not successful in young children, one focus of the presented intervention is on the cervical muscles as the easily accessible peripheral input of TCC [10]. With physiotherapeutic treatment of the muscle, including self-massage and stretching, the tenderness of the muscles is reduced which in our clinical experience leads to a decrease of headache frequency and/or intensity.

**Fig. 3:**
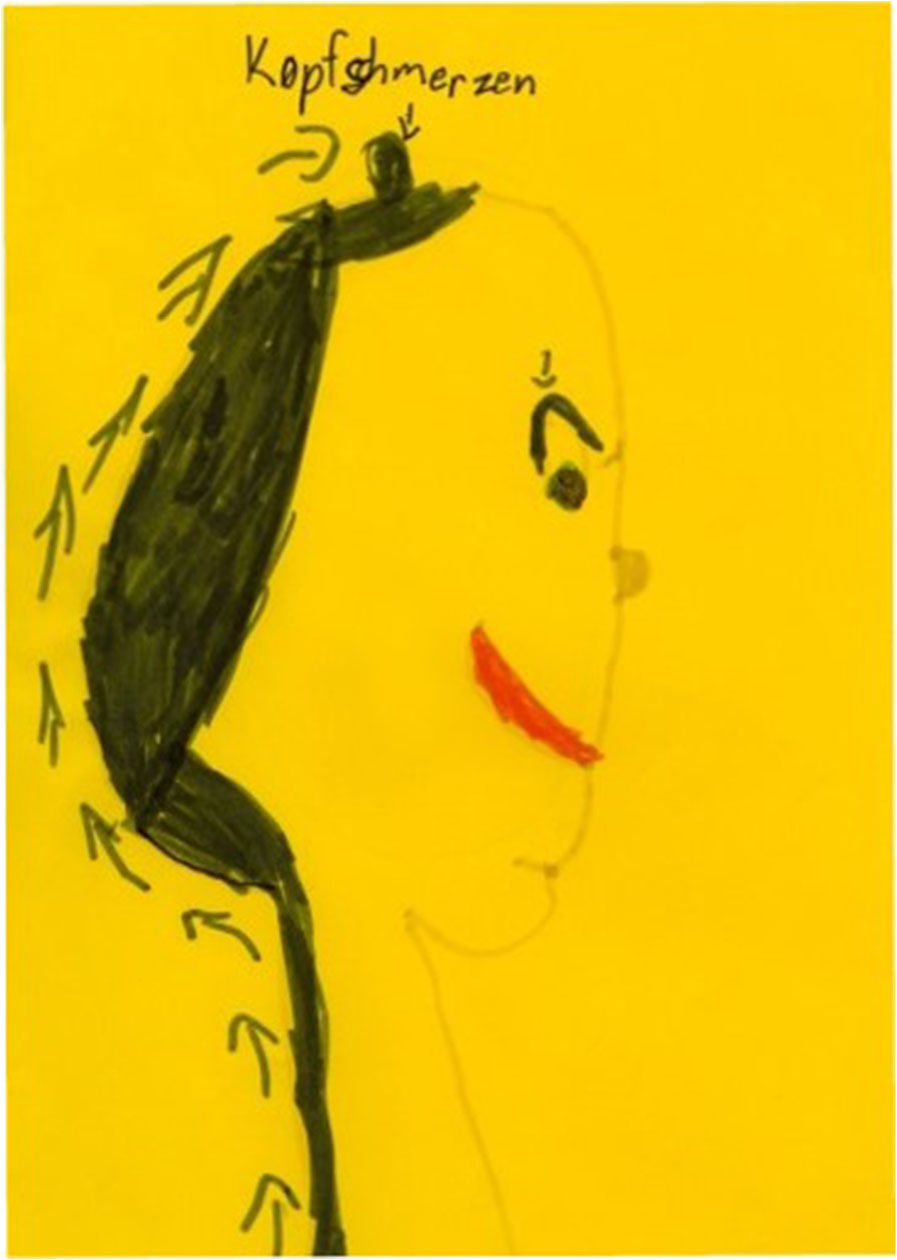
Drawing of his headache by a 9-year-old child

In addition, progressive muscle relaxation, which was shown to be effective in adults with migraine [13] was adapted for young children and is integrated in the psychological module of the presented intervention. Psychologists also screen for psychological stress and psychiatric comorbidities which are more frequent in patients with migraine compared to healthy persons [14]. The treated children are asked to draw their headache to visualize individual symptoms of migraine (Fig. 3) [15].

#### Measures

All outcome parameters are assessed via the dedicated smartphone application ‘moma app’, which is filled in by the parents over the 9 months study duration (Fig. 2).

The app contains the following features:

1. Headache diary (based on established diaries [16, 17]) including a daily reminder function (missed entries days can be catched up within 2 weeks), with daily assessment of the following items

- Presence of headache (yes/no)
- Headache intensity (numeric rating scale 0–10 presented as a slider)
- Headache duration (slider from 0 to 24 h)
- Missed school (yes/no)
- Inabilty to perform leisure activities (yes/no)
- Use of headache medication (yes/no, tool for recording medication and dose)

2. Questionnaires are presented within the app at the start and end of the baseline (weeks − 12 and 0), at the end of the intervention (week 12) and at the end of the follow-up (week 24). The following questionnaires are used:

- PedMIDAS (Pediatric Migraine Disability assessment score) [18]
- KINDL [19] (German tool for assessing pediatric quality of life).

3. At the time points used for the questionnaires, the smartphone app also asks for

- typical triggers of headache in the last 3 months (within the categories: school stress, family problems, conflicts with friends, noise, sport, inactivity, weather, lack of sleep, drinks containing caffeine, reduced fluid intake, high use of electronic media, infection, others)
- headache prophylactic measures taken in the last 3 months (within the categories: physiotherapy, trigger point therapy, osteopathy, acupuncture, homeopathy, alternative practitioner, naturopathy, peppermint oil, magnesium, vitamin D, medication, others)

Additionally to the ‘moma App’, pediatricians in practice and social pediatric centers use the ‘moma website’ (www.moma-migraine.de) [1] to document the individual clinical presentation and characteristics of the child, for example individual migraine symptoms, important aspects of the medical history, results of the neurological examination, psychosocial factors and the therapeutic recommendations given to the family. Furthermore, they estimate the complexity of the disease for each child using the ‘moma complexity signature’, which has been developed by our group specifically for this study based on the bio-psycho-social model (see Fig. 3 for time points of data acquisition).

### Outcome parameters

All outcome parameters are assessed via the parents’ entries in the ‘moma App’. For all outcome parameters, the difference from baseline to follow-up is measured and compared between the two groups.

The primary outcome parameter is the number of headache days per 12 weeks (84 days). Our hypothesis is that the reduction of headache days from baseline to follow-up will be at least 2 days larger for the group with interdisciplinary multimodal intervention in social pediatric centers (intervention group B) compared to the group with basic treatment in pediatric practices (intervention group A).

Secondary outcomes are:

1. Number of responders, defined as patients with a reduction of more than 50% of headache days per 12 weeks from baseline to follow-up;
2. Headache intensity (averaged over headache days per 12 weeks);
3. Number of days missed at school due to headache per 12 weeks;
4. Number of days with cancelling of leisure activities due to headache per 12 weeks;
5. Headache-related disability (PedMIDAS questionnaire) [18],assessed every 3 months;
6. Quality of life (KINDL questionnaire, every 3 months) [19, 20];
7. Number of days with intake of acute medication because of headache per 12 weeks.

### Data analysis plan

The statistical analysis is performed according to the CONSORT Statement recommendations for cluster-randomized trials [20]. In the intention-to-treat analysis all patients having started to use the ‘moma App’ are included. Primary endpoint is the number of headache days per 12 weeks (84 days).

Two forms of analysis are performed:

1. Intention-to-treat analysis (“Full Analysis Set”)

All patients who started to use the electronic headache diary (moma app) and fulfill the baseline criteria (see below) are included in the intention-to-treat analysis. Patients who have less than three headache days during the 12 weeks of baseline or from whom less than 33% of the days of the headache diary are filled in during baseline are excluded and are not part of the intention-to-treat analysis.

2. On-treatment analysis (“per protocol set“)

Patients who additionally completed all 4 study visits at the pediatric practices and (only in the intervention group) the three visits at the social pediatric centers and provided electronic headache diary data with less than 33% of the days missing during the intervention and follow-up phase are included in the per protocol analysis.

For headache diary data, missing data are treated as follows. If less than 33% of the days are missing, the missing data is replaced by the mean values of the existing data. If 33 to 66% of the headache diary data is missing, a modified last observation carried forward (LOCF) approach will be performed for the intention to treat analysis. To this end, missing days are replaced by the mean values of the the last 3-month period that has less than 33% missing data. If more than 66% of the data of the headache diary data is missing, a simple LOCF principle will be applied. For this, the data of the entire 3-month period will be replaced by the mean value of the last 3-month period with less than 33% missing data.

If one or more items of the PedMIDAS (6 items) is missing, the scale will be excluded from statistical analysis of this patient at this time point because it does not retain sufficient clinical informative value.

The KINDL questionnaire (4 subscales with 4 items each) allows one missing item per subscale which is replaced by the mean value of the other items of the subscale [19, 20].

For the primary end point (number of headache days per 12 weeks) a mixed linear regression model is performed with the difference of headache days per 12 weeks from baseline to follow-up as dependent variable, the intervention in a social pediatric center (yes/no) as independent variable and random effects for the different social pediatric centers. Age, sex and headache frequency per 12 weeks at baseline are included as possible confounders. Quality of life (KINDL) and migraine related disability (PedMIDAS) at baseline are possible effect modifying elements and therefore integrated in the model as interaction effects. The significance level α is set at < 0.05. The same model is used for analysis of the secondary outcome parameters.

## Discussion

### Ethics, trial registration and data management

The study was approved by the local ethics committee at the Ludwig-Maximilians-University Munich (number 18–804, leading ethics committee) and approval was confirmed by all the other German ethics committees relevant for the study (*n* = 15). The study was retrospectively registered on 27 April 2021 in the WHO approved German Clinical Trials Register (number DRKS00016698). Data protection is established according to the European data protection regulation 2016/679. Only the data necessary for study conduction and analysis is obtained via the smartphone app (from the parents) and via the moma website (from the pediatricians in practice and from the social pediatric centers). The study data is saved on a dedicated server located at the University of Hof, Germany. Data is pseudonymized at the time point of collection using a hash-code based on the patient’s name and birth date. This hash-code is only used to link data from the same patients on the server. It does not allow back-tracing of the patient’s name or birth date. The data will be stored for ten years.

### Importance and dissemination

Migraine is a prevalent and disabling disease in childhood. Therapeutic options are scarce or not well tolerated by the children or their parents. Studies about the efficacy of non-invasive, non-pharmaceutical interdisciplinary treatment in young children with focus on the association of muscle tenderness and migraine do not exist so far. Hence, the presented intervention study is of high interest not only for the young patients but also for the pediatric and scientific communities as well as for the health systems worldwide. Therefore, we plan to publish the results of the multimodal intervention study in international scientific medical journals with a high distribution rate among pediatricians and neuropediatricians. As the topic is also relevant for health politics, the results will also be published at the homepages or newsletters of relevant medical professional societies. The study outcomes will also be discussed at national and international congresses for (Neuro-) Pediatrics.

### Strengths and limitations

The first strength of the study is the possible positive impact of the results for the participating child with migraine and its family. The second strength is the randomized controlled design with planned nationwide participation of pediatricians in practice and of interdisciplinary social pediatric centers. The third strength is the clinical and political importance of the study subject: interdisciplinary intervention programs for children with migraine are mandated but efficacy studies in this age group are lacking.

A limitation pertains to health system differences between countries, reducing generalizability of study results. The realization of study might be cumbersome because of challenges for pediatric practices related to the current COVID-19 pandemic.

## Data Availability

The datasets used and analysed during the current study are available from the corresponding author on reasonable request.

## Abbreviations

α: Significance level
App: Smartphone application
approx: Approximately
COVID-19: Coronavirus disease 2019
G-BA: Gemeinsamer Bundesausschuss = Federal Joint Committee
ICC: Intra-class correlation coefficient
ICD-10: International Classification of Diseases 10th revision
ICHD-3: International Classification of Headache Disorders 3rd edition
IQ: Intelligence quotient
KINDL: Name of a German tool for assessing pediatric quality of life
LOCF: Last observation carried forward
moma: Modules on migraine activity = name of the study
n: Number
NSAID: Nonsteroidal antiinflammatory drugs
PedMIDAS: Pediatric Migraine Disability Assessment Score
TCC: Trigemino-cervical complex
WHO: World Health Organization
